# Epidemiology, risk factors, and prevention of animal bites injuries in Slovakia: an 18-year epidemiological analysis

**DOI:** 10.3389/fpubh.2026.1868624

**Published:** 2026-09-09

**Authors:** Ahmad Gharaibeh, Mahmoud M. Gharaibeh, Róbert Čellár, Marek Lacko, Dávid Sokol, Simona Miškárová, Martina Hrubovčák Tejová, Kvetoslava Rimárová

**Affiliations:** 1 Department of Orthopaedics and Musculoskeletal Trauma, Louis Pasteur University Hospital, Košice, Slovakia; 2 Univerzita Pavla Jozefa Safarika v Kosiciach Lekarska fakulta, Košice, Slovakia; 3 Department of Dentistry, Princes Basma Teaching Hospital, Irbid, Jordan; 4 Department of Public Health and Hygiene, Faculty of Medicine, Pavol Jozef Šafárik University, Košice, Slovakia

**Keywords:** animal exposures, epidemiology, rabies prevention, Slovakia, Zoonoses

## Abstract

**Background:**

Animal bite injuries, predominantly from dogs, rank among the top 15 causes of nonfatal injuries globally. Since 2006, negligible number of cases of rabies were declared in Slovakia, lacks comprehensive national data on bite demographics and outcomes.

**Methods:**

This Epidemiological study analysed 14,978 patient records (2006–2023) obtained from Slovak public health databases. Statistical analyses, including chi-square tests and linear regression, were used to evaluate trends in bite incidence, vaccination coverage, and associated risk factors.

**Results:**

Between 2006 and 2023, 14,978 individuals sought treatment for animal exposures, with 88% of exposures attributed to domestic animals, primarily dogs (78.9%). Children aged 5–9 years and adults aged 25–34 years represented the highest-risk groups. The authors observed a significant downward trend in bite incidence over time (*β* = −0.507, *p* = 0.002), with hands being the most commonly affected sites (41%). No human rabies cases were reported after 1990, and nationwide animal vaccination programs led to the near elimination of rabies by 2006. However, the authors recorded a 50% increase in bite incidents in the post-lockdown period (2022–2023), predominantly affecting males. The analysis demonstrated a significant negative temporal trend, with animal bite incidence decreasing by approximately 0.51 cases per 100,000 population per year (*β* = −0.507, 95% CI: −0.82 to −0.19, *p* = 0.002).

**Conclusion:**

Slovakia’s rabies control program represents a major public health success; however, animal exposures—particularly dog bites—remain a persistent problem. A temporary decline in bite incidence was observed during COVID-19 lockdowns, followed by a marked post-pandemic increase. Targeted prevention strategies should focus on high-risk populations, promote responsible pet ownership, and address emerging post-pandemic trends.

## 1 Introduction

Animal bite injuries represent a substantial global public health burden, with dogs responsible for approximately 76%–94% of reported cases worldwide (1–3). Paediatric populations are disproportionately affected, experiencing higher incidence rates and more severe injuries compared with adults. Nevertheless, considerable regional variation exists in bite severity, clinical outcomes, and prevention strategies (4–6). In Switzerland, approximately 10,000 individuals are bitten by dogs each year (4). In the United Kingdom, reported dog bite incidents more than doubled between 1998 and 2018, with associated healthcare and societal costs reaching £70.8 million in 2017–2018 (4). Between 1995 and 2016, the number of fatalities resulting from dog bite injuries increased across Europe, with 45 deaths reported in 2016 (5). In the United States, dog bite injuries represent one of the leading causes of non-fatal traumatic injuries among children and adolescents (7). A Canadian study further identified paediatric age as a significant risk factor for dog bite injuries, highlighting persistent gaps in knowledge regarding their occurrence and associated determinants (8). Overall, existing evidence indicates that dog bite injuries constitute a major public health concern, not only due to the high number of emergency department visits but also because of their potential long-term psychological consequences, particularly among children (9).

Since 2006, negligible number of cases of rabies were declared in Slovakia (10, 11), offers a unique setting for studying the epidemiology of animal bite injuries in a rabies-free environment. Despite successful rabies elimination, dog bites remain a significant public health concern and continue to rank among the top 15 causes of nonfatal injuries in Western Europe (1–3, 12). Epidemiological evidence highlights age-specific risk patterns. A study from Slovenia reported that children aged 1–9 years accounted for 35.8% of dog bite cases, whereas those aged 10–19 years represented 64.2% (13). In contrast, a study from Chile documented the highest incidence among adults aged 40–64 years, underscoring the need for population-specific prevention programmes (14). According to a comprehensive literature review, the head and neck region remains the most frequently affected anatomical site in animal bite injuries, particularly among younger children (14, 15). This finding reinforces the importance of age-appropriate preventive interventions and increased supervision. Although head and neck injuries are often regarded as the most severe outcomes, bites affecting the limbs and torso also warrant attention due to their potential long-term physical and psychological consequences for children (8, 16). In the United Kingdom, one study reported that 62.3% of dog bite incidents did not require medical treatment (15). In Australia, mortality from dog bite injuries is approximately 50% lower than in Europe, despite similar overall bite incidence rates. Notably, infants constitute a substantial proportion of fatal dog bite victims in both regions (5).

Currently, Slovakia lacks a comprehensive national study addressing animal bites and potentially dangerous contacts with animals across all age groups, particularly among children, adolescents, and older adults.

1. Aim of the work:

- The primary aim of this study was to analyse data from the Slovak national public health database to support the development of targeted prevention programmes for children and adolescents at increased risk of animal bite injuries in Central Europe.
- Additionally, this study examined the anatomical distribution of injuries, sex-specific differences, mortality, the occurrence of rabies in humans and animals, types of potentially dangerous animal contacts, and adherence to post-exposure anti-rabies vaccination protocols. The findings of this study may contribute to future research and inform the development of effective prevention strategies aimed at investigating, controlling, and ultimately preventing animal bite injuries at both national and regional levels and to prevent animal exposure, particularly involving dogs. Furthermore, the study sought to assess risk factors associated with interactions between domestic animals and humans, with particular attention to households with children.
- The specific objectives of this study were to identify age groups at the highest risk of animal bite injuries with suspected rabies exposure.
- Critical gap addressed: No national study has comprehensively examined age-specific risks, vaccination effectiveness, adherence to post-exposure prophylaxis (PEP) protocols, or post-lockdown trends in animal bite incidents. This study addresses these gaps by providing the first national epidemiological analysis of animal bite injuries and suspected rabies exposures over an 18-year period, including demographic and anatomical patterns, rabies elimination efforts, PEP adherence, and post-pandemic changes in bite incidence.

## 2 Materials and methods

### 2.1 Data sources

This Epidemiological study analysed 14,978 anonymised records obtained from the Public Health Authority of the Slovak Republic and the State Veterinary and Food Administration of the Slovak Republic, covering the period 2006–2023. The database included cases of suspected rabies exposure resulting from interactions with both domestic and wild animals.

Recorded exposures comprised animal bites, scratches, drooling, and other forms of potentially hazardous contact, including rubbing, licking, direct physical contact, inhalation exposure, contact with animal excreta (faeces or urine), and contact with animal fur. The dataset also contained information on the medical care provided at healthcare facilities throughout Slovakia during the study period is complete and standardized. Partial records were available for the years 1987–2005, while no data were available prior to 1987. The following missing variables were recorded: type of exposure (bite, scratch, drooling, or other potentially hazardous contact), anatomical location of injury, and animal species involved (dogs, cats, other domestic animals, and wild animals).

### 2.2 Variables

The dataset included the following variables:

- Demographic characteristics: age and sex
- Type of exposure: bite, scratch, drooling, or other potentially hazardous contact
- Anatomical location of injury
- Animal species involved: dogs, cats, other domestic animals, and wild animals
- Vaccination status of the animal: complete, incomplete, or missing records
- Occurrence of rabies in domestic and wild animals
- Animal-related fatalities, stratified by age group and animal type

### 2.3 Age classification

Age groups were categorised according to the Slovak medical paediatric classification as follows:

- Children (0–19 years)

**(**The Slovak Pediatric Association provides specialized care for children up to the age of 19).

- <1 year
- 1–4 years
- 5–9 years
- 10–14 years
- 15–19 years

According to the UN Convention on the Rights of the Child as adopted in the Slovak Republic, a child is defined as any individual under the age of 19 years, unless otherwise stipulated by applicable law. This study used the following age categories: infants (<1 year), young children (1–4 years), children (5–9 years), pre-adolescents (10–14 years), and adolescents (15–19 years). These groupings are widely recognized in healthcare, education, and developmental psychology.

- Adolescents: 20–24 years
- Adults:
- 25–34 years
- 35–44 years
- 45–54 years
- 55–64 years
- ≥65 years

### 2.4 Ethical considerations

Due to the epidemiological and anonymized nature of the data, the authors waived the requirement for informed consent. All study procedures were conducted in accordance with relevant ethical standards and applicable regulations. The data were provided pursuant to § 15(1) of Act No. 211/2000 Coll. on Free Access to Information. Ethical approval was not required, as the data were obtained directly from the Public Health Authority of the Slovak Republic and the State Veterinary and Food Administration of the Slovak Republic in compliance with this legal provision.

### 2.5 Statistical analysis

The study period extended from 1 January 2006 to 31 December 2023, with complete and standardized data. In addition, partial historical records were available for the years 1987–2005, comprising a total of 51,208 cases. These historical data were used solely for descriptive and contextual analyses. The authors analyzed categorical variables (e.g., anatomical location of injury stratified by age group) using the chi-square (χ^2^) test. Linear regression analysis was applied to evaluate temporal trends in animal bite incidence over time, with incidence rates expressed as cases per 100,000 population. For statistical analyses, patients were stratified into the following age groups: paediatric population (0–19 years), adults (20–64 years), and older adults (>65 years). The authors performed all statistical analyses using standard epidemiological methods and defined statistical significance as a *p*-value < 0.05.

## 3 Results

**Figure 1:**
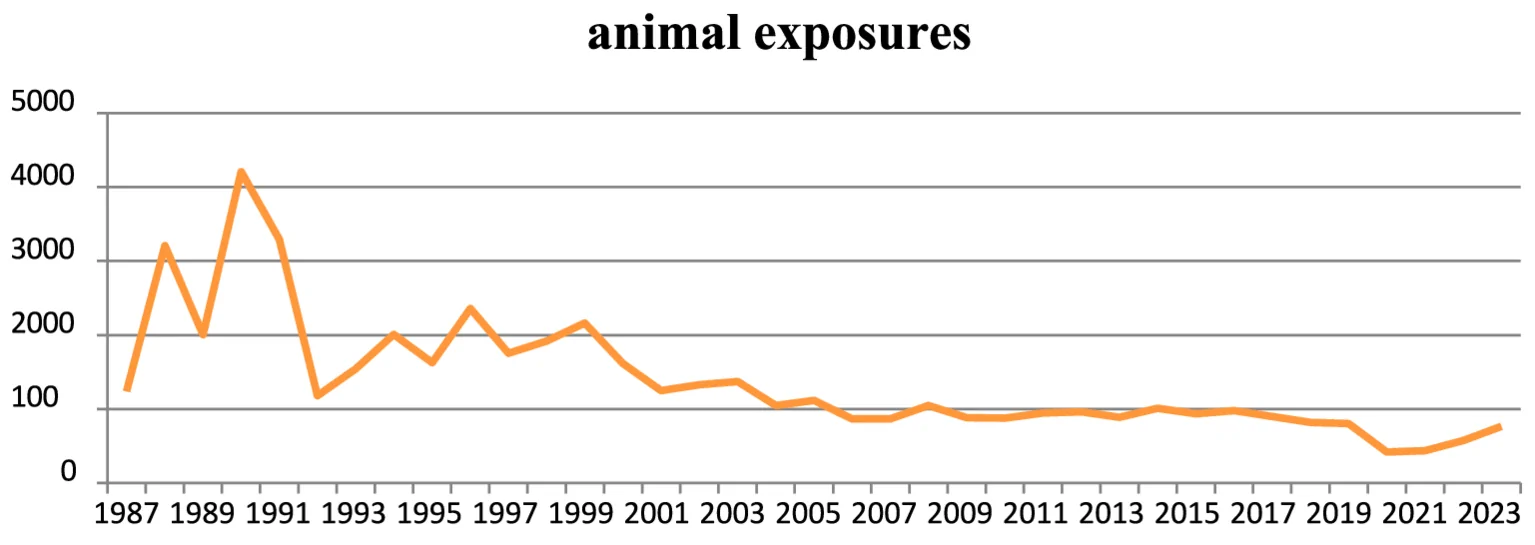
Annual incidence rate of reported animal exposure incidence in Slovakia (1987–2023).

A total of 51,208 individuals received medical treatment for animal bites injuries or contact with animals suspected of rabies infection in Slovakia between 1987 and 2023 (Figure 1). No cases of human rabies were reported between 1991 and 2023. The last confirmed case of human rabies occurred in 1990, with the previous case reported in 1977.

A detailed analysis was performed on 14,978 cases recorded between 2006 and 2023, for which complete and standardized records were available from the Public Health Authority of the Slovak Republic. The majority of animal exposure incidents during this period were attributed to dogs, followed by cats, with wild animals accounting for a smaller proportion of cases.

A statistically significant variation in the distribution of animal species (Home bits and wild) involved in human exposures was observed over time [χ^2^(34) = 178.239, *p* < 0.001], indicating a temporal shift in exposure patterns (Figure 1).

Overall, the incidence rate of reported animal attacks and suspected rabies exposures in Slovakia has declined over the past 37 years. The authors recorded the highest incidence in the early 1990s, with 4,208 cases in 1990 and 3,294 cases in 1991 (Figure 1). The authors observed a marked reduction thereafter.

During the COVID-19 lockdown period (2020–2021), the number of reported animal attacks temporarily decreased, with 419 cases in 2020 and 435 cases in 2021. However, the authors found that this decline was followed by a substantial post-pandemic increase, with animal bite incidents rising by approximately 50% in 2022–2023, indicating a renewed upward trend after the easing of public health restrictions.

A statistically significant association was observed between animal type and year, indicating that the distribution of animal species involved in bite incidents varied significantly over time (Table 1; Figure 1).

**Table 1:** Chi-square test: distribution of animal types across years.

Test	χ^2^ value	df	*p*-value
Animal_type × Year	178.239	34	<0.001

To further assess temporal trends, a linear regression analysis was performed with calendar year as the independent variable and the incidence of animal bites per 100,000 population as the dependent variable. The analysis demonstrated a significant negative temporal trend, with animal bite incidence rate decreasing by approximately 0.51 cases per 100,000 population per year (*β* = −0.507, 95% CI: −0.82 to −0.19, *p* = 0.002) (Table 2).

**Table 2:** Linear regression analysis of temporal trends in animal bite incidence in Slovakia (2006–2023).

Predictor	B (95% CI)	β Beta	*p*-value	R^2^
Year	−0.507 (−0.82 to −0.19)	−0.712	0.002	0.508

The regression model explained 50.8% of the variance in annual incidence rates (R^2^ = 0.508), indicating a temporal association between year and animal bite incidence.

Linear regression analysis demonstrated a statistically significant negative temporal trend in animal exposure incidence over time. Incidence rates were calculated per 100,000 population using annual national census estimates (Table 2).

Over the 18-year study period (2006–2023), the mean annual number of animal bite incidents was 764.6 (95% CI: 682.5–846.7). During the pre-pandemic period (2006–2019), the mean was 796.1 bites per year. A marked decline was observed during the COVID-19 pandemic trough (2020–2021), with a mean of 393.5 bites per year, representing a 50.6% reduction compared with the pre-pandemic mean. In the post-pandemic period (2022–2023), the mean increased to 636.0 bites per year, a 61.6% rise from the pandemic trough, although this remained 20.1% below the pre-pandemic average. By 2023, bite incidents had recovered to 725 cases, still 8.9% lower than the pre-pandemic mean of 796.1. Annual distribution of animal exposure incidents by contact type recorded in the Slovak national public health database, 2006–2023. Percentages represent the proportion of each contact type within the total exposures for that year. Bites consistently accounted for >90% of all reported exposures across the study period (Table 3).

**Table 3:** Annual number of animal bite incidents and other animal exposures in Slovakia and exposure rate per 100000, 2006–2023.

Year	Population of Slovakia	Total exposures	Bite *n* (%)	Scratch *n* (%)	Drooling *n* (%)	Another contact *n* (%)	Exposure rate per 100,000
2023	5,795,199	765	725 (94.8%)	36 (4.7%)	1 (0.1%)	3 (0.4%)	13.2
2022	5,643,453	574	547 (95.3%)	11 (1.9%)	1 (0.2%)	17 (3.0%)	10.17
2021	5,447,621	435	413 (94.9%)	13 (3.0%)	0 (0.0%)	9 (2.1%)	7.98
2020	5,459,642	419	374 (89.3%)	39 (4.9%)	1 (0.1%)	5 (0.6%)	7.67
2019	5,457,873	803	758 (94.4%)	39 (4.9%)	1 (0.1%)	5 (0.6%)	14.71
2018	5,450,421	819	781 (95.4%)	33 (4.0%)	0 (0.0%)	4 (0.5%)	15.03
2017	5,433,203	898	847 (94.3%)	40 (4.5%)	3 (0.3%)	8 (0.9%)	16.53
2016	5,430,795	978	934 (95.5%)	33 (3.4%)	1 (0.1%)	7 (0.7%)	18.01
2015	5,427,842	937	893 (95.3%)	34 (3.6%)	4 (0.4%)	3 (0.3%)	17.26
2014	5,424,569	1,010	936 (92.7%)	57 (5.6%)	6 (0.6%)	3 (0.3%)	18.62
2013	5,421,084	888	815 (91.8%)	54 (6.1%)	2 (0.2%)	15 (1.7%)	16.38
2012	5,417,402	963	876 (91.0%)	55 (5.7%)	2 (0.2%)	20 (2.1%)	17.78
2011	5,413,442	948	850 (89.7%)	61 (6.4%)	2 (0.2%)	35 (3.7%)	17.51
2010	5,409,149	879	800 (91.0%)	43 (4.9%)	1 (0.1%)	25 (2.8%)	16.25
2009	5,404,437	883	823 (93.2%)	41 (4.6%)	2 (0.2%)	17 (1.9%)	16.34
2008	5,399,345	1,047	979 (93.5%)	52 (5.0%)	4 (0.4%)	10 (1.0%)	19.39
2007	5,394,226	867	804 (92.7%)	39 (4.5%)	6 (0.7%)	18 (2.1%)	16.07
2006	5,393,637	865	805 (93.1%)	44 (5.1%)	1 (0.1%)	15 (1.7%)	16.04

**Figure 2:**
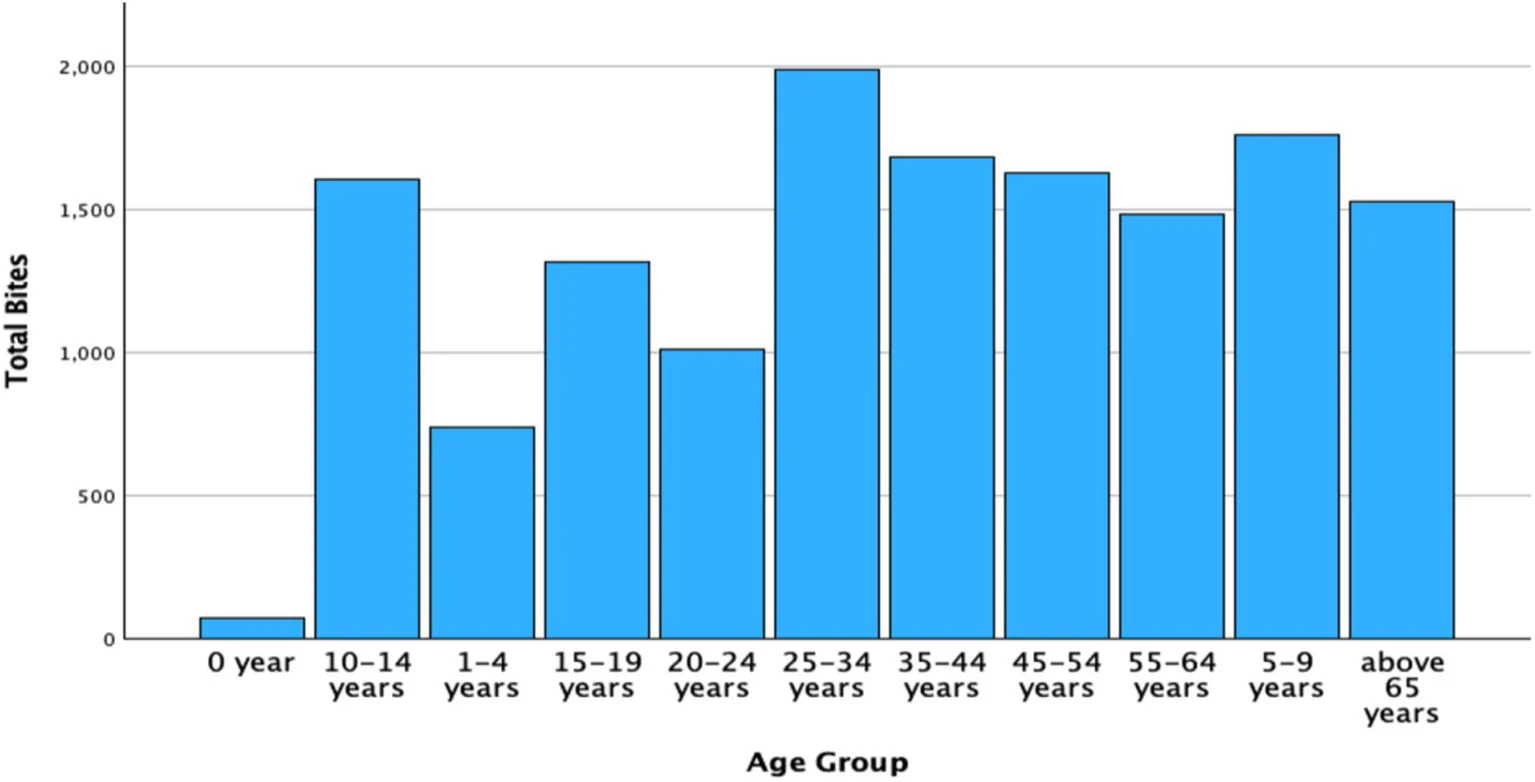
Total number of animal bite incidents by age group.

**Figure 3:**
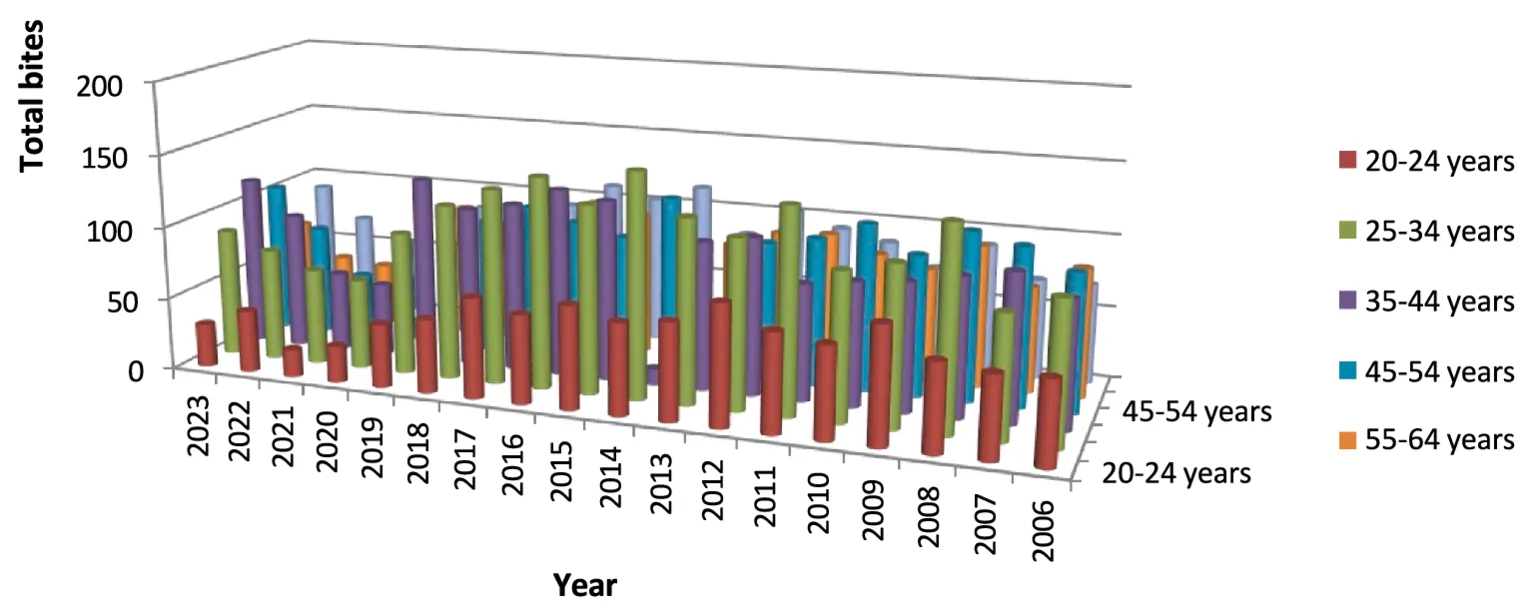
All animal exposures in adult age group.

**Figure 4:**
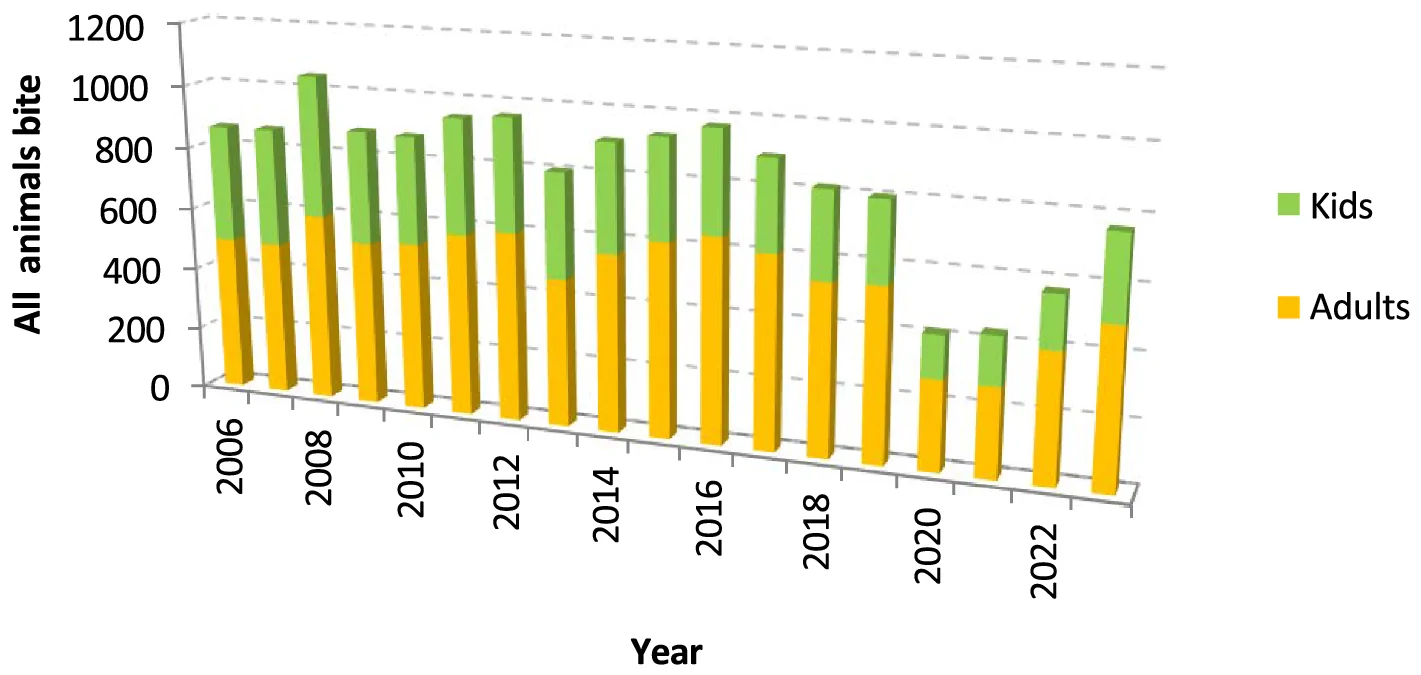
Contact with animals in kids and adults.

Significant differences were observed among adult age categories (χ^2^ = 91.456, *p* < 0.01). Individuals aged 25–34 years represented the most frequently affected group, whereas the lowest number of animal bite incidents was recorded among individuals aged ≥65 years (Figures 2–4).

**Figure 5:**
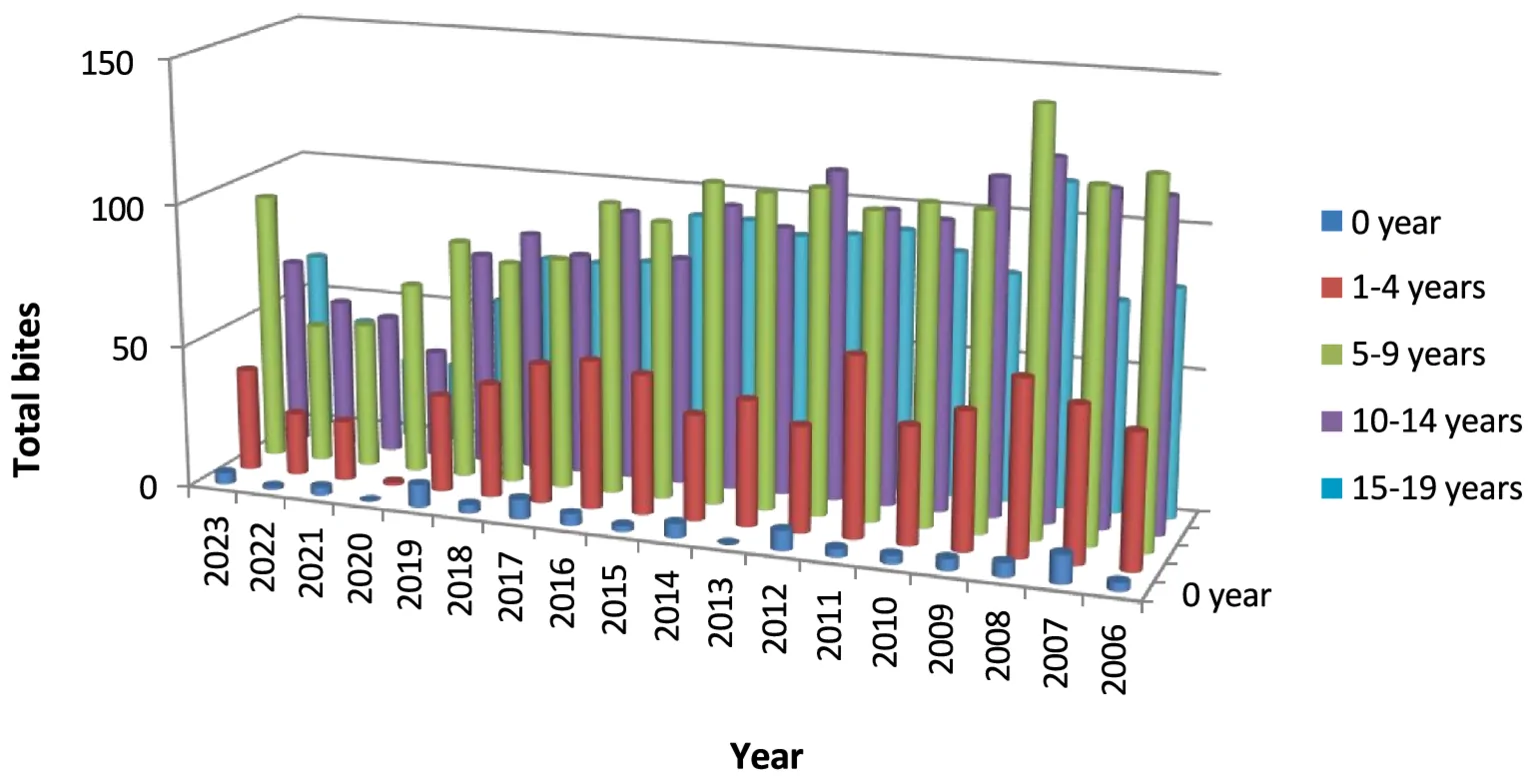
All animal exposures in kids’ age groups.

Among children (0–19 years) and young adults (20–24 years), the incidence of animal bite injuries varied significantly by age group (χ^2^ = 1501.05, df = 5, *p* < 0.01). The highest number of exposures was observed in children aged 5–9 years, while the lowest incidence occurred among infants under 1 year of age (Figures 2–5).

Overall, the most affected age group by counts was 25–34 years, followed by individuals aged 35–44 years and 45–54 years. Although adult populations accounted for the majority of cases, paediatric bite injuries represented a substantial proportion, highlighting children as a persistently vulnerable group (Figures 2–5).

**Figure 6:**
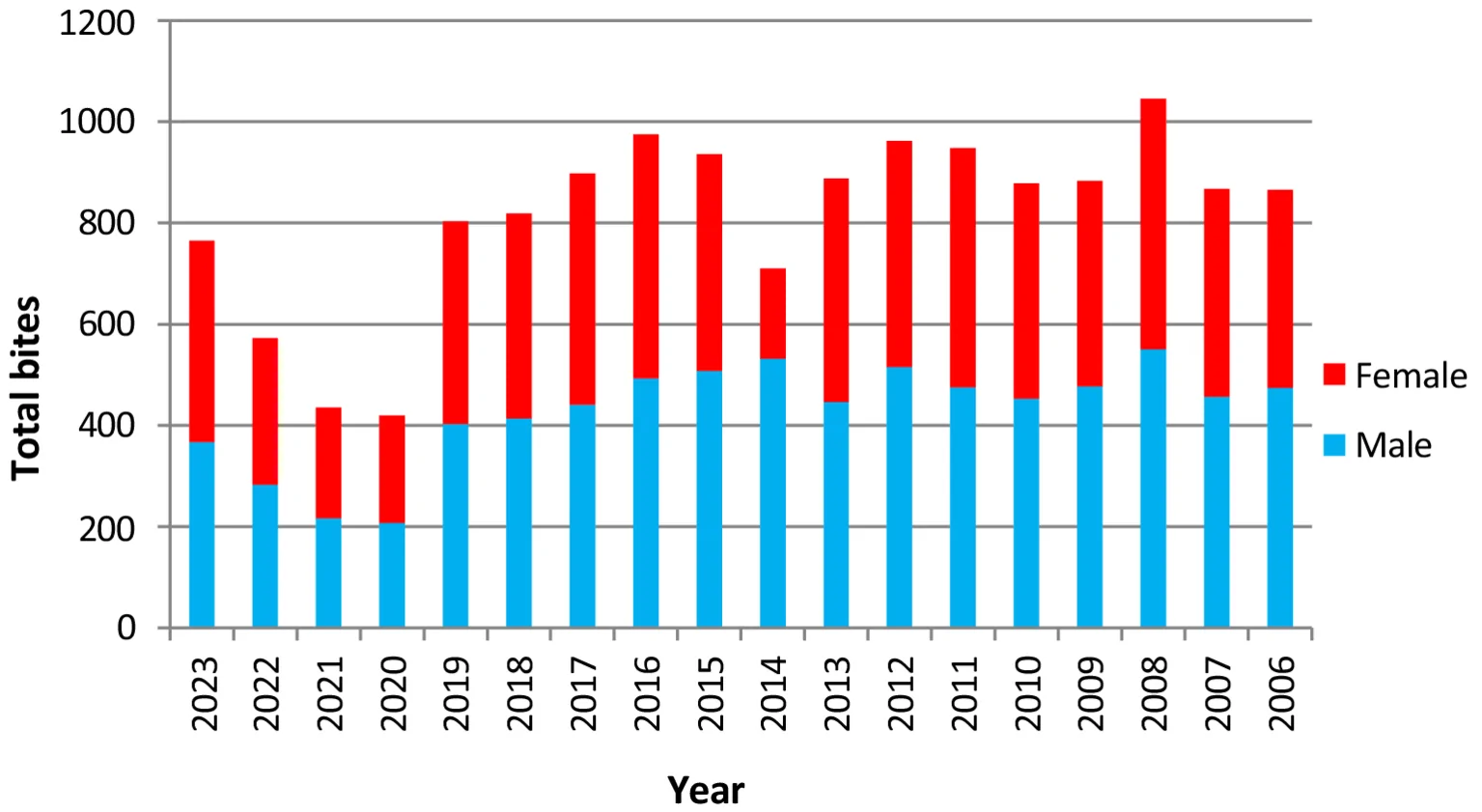
All animal exposures in both sexes.

The authors observed a statistically significant difference between males and females in the occurrence of animal bite and scratch injuries, with males accounting for 47.42% of cases (χ^2^ = 51.034, *p* < 0.01). The authors also identified a significant difference between children and adults, with adults experiencing a higher number of animal bite incidents (χ^2^ = 777.04, *p* < 0.01) (Figure 6).

**Figure 7:**
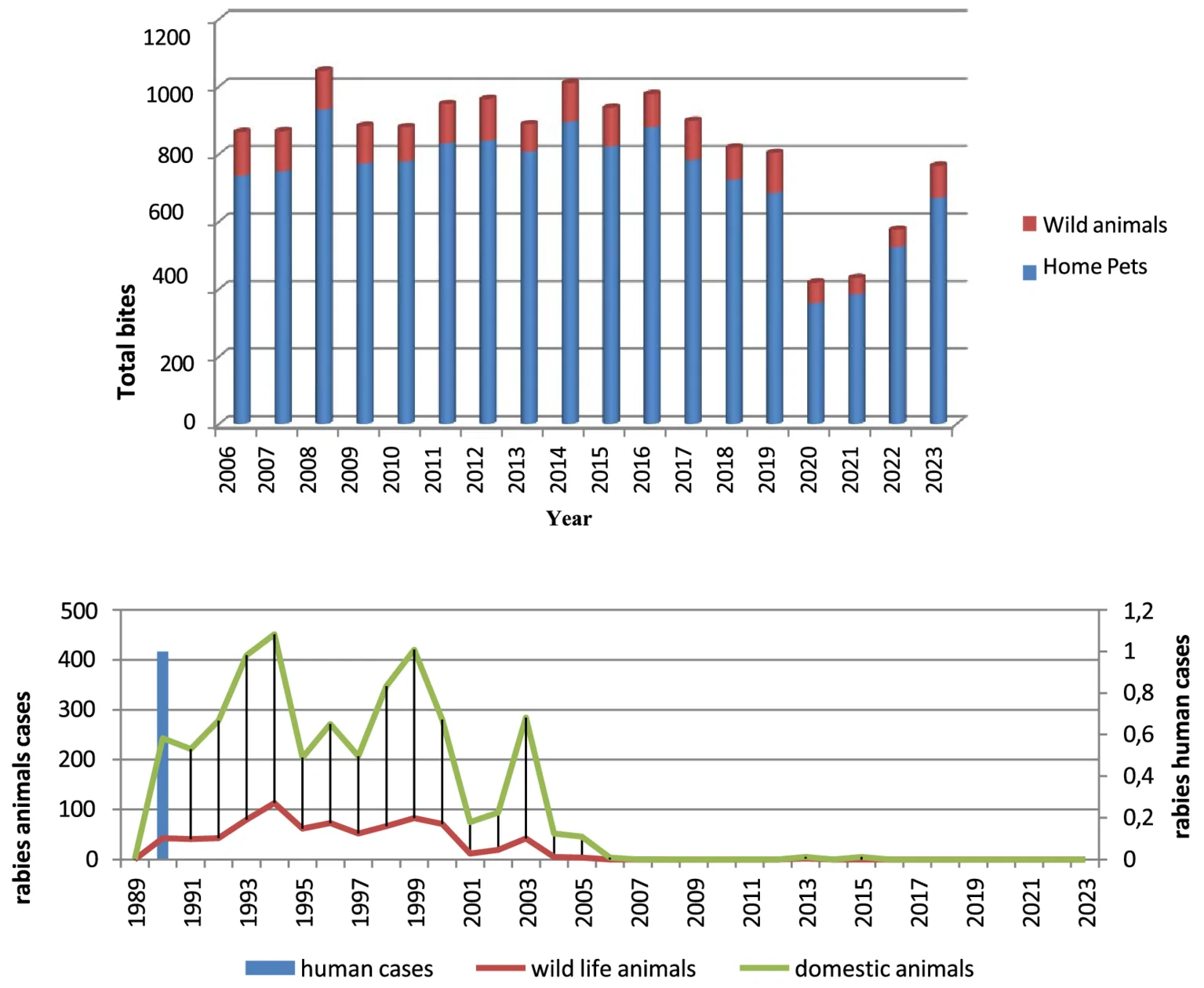
Incidence of animal and human rabies cases in Slovakia.

The authors observed a statistically significant difference between the number of rabies-infected domestic and wild animals reported between 1989 and 2023 (χ^2^ = 2021.071, *p* < 0.01) (Figure 6). The last confirmed case of human rabies in Slovakia occurred in 1990 and resulted from a scratch inflicted by a stray cat. No human rabies cases have been reported since 1990. Furthermore, following the implementation of a nationwide animal vaccination programme, negligible number of cases of animal rabies have been reported in Slovakia since 2006 (Figure 7).

An analysis of the relationship between vaccination status—complete post-exposure prophylaxis (PEP), incomplete PEP, and no vaccination—and the occurrence of human rabies revealed no statistically significant association (*p* > 0.05). Importantly, no human rabies cases were recorded between 2006 and 2023, regardless of vaccination status.

Among the 14,978 individuals included in the detailed analysis, 72.3% (11,109/14,978) received complete PEP, 1,395 individuals received partial vaccination, and 2,258 individuals were unvaccinated at the time of exposure. Despite these exposures, no cases of human rabies occurred, including among unvaccinated individuals.

Between 2006 and 2023, a total of 45 adult fatalities related to animal bite injuries were recorded in Slovakia (34 men and 11 women). No fatalities were reported among children or adolescents during the study period.

A comparison of fatal outcomes by sex revealed a statistically significant difference, with male fatalities occurring more frequently than female fatalities (χ^2^ = 6.737, *p* < 0.01). Men accounted for 75.6% of all deaths (34/45), whereas women represented 24.4% (11/45) of fatal cases.

**Figure 8:**
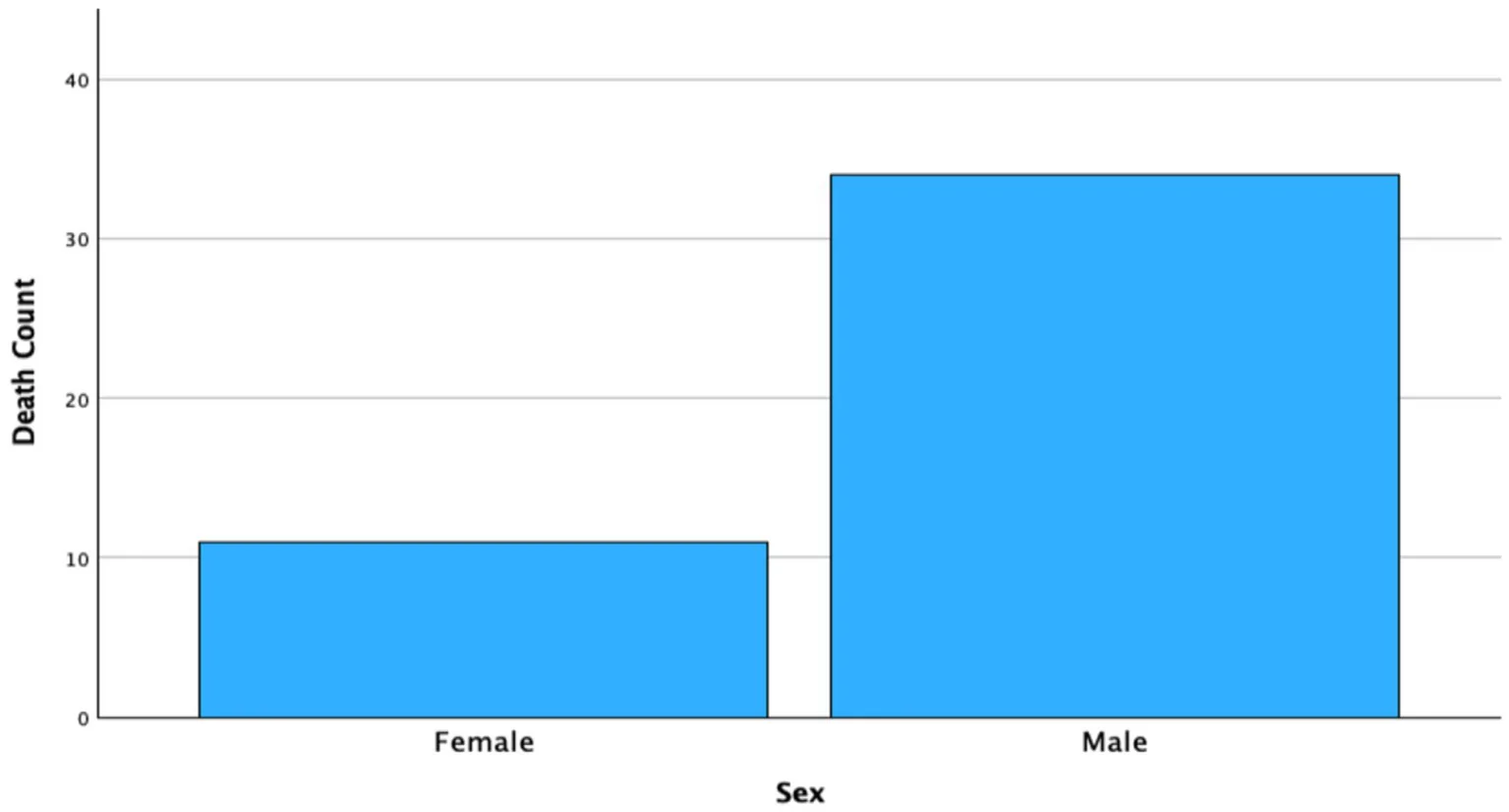
Fatalities by sex.

The majority of fatal outcomes were observed among individuals aged ≥65 years, indicating a higher mortality burden in older age groups (Figure 8; Table 3).

A chi-square test indicated a trend toward statistical significance in the association between sex and fatal outcome, with a higher number of deaths observed among males; however, this association did not reach conventional levels of statistical significance (χ^2^ = 3.076, *p* = 0.079). Additional data would be required to confirm this relationship (Table 4).

**Table 4:** Chi-square test: fatality by sex.

Variable interaction	χ^2^ value	df	*p* value
Sex × Outcome	3.076	1	0.079

Significant differences were observed across age categories, with the highest number of fatalities occurring in individuals aged ≥65 years, indicating increased vulnerability in older populations. During the study period (2006–2023), a total of 45 adult deaths related to animal bite injuries were recorded. Of these, 33 fatalities (73.3%) resulted from dog bites, while 12 fatalities (26.7%) were associated with wild animal exposures. This difference was statistically significant (χ^2^ = 6.737, *p* < 0.01), indicating that dog bites accounted for the majority of fatal outcomes.

**Figure 9:**
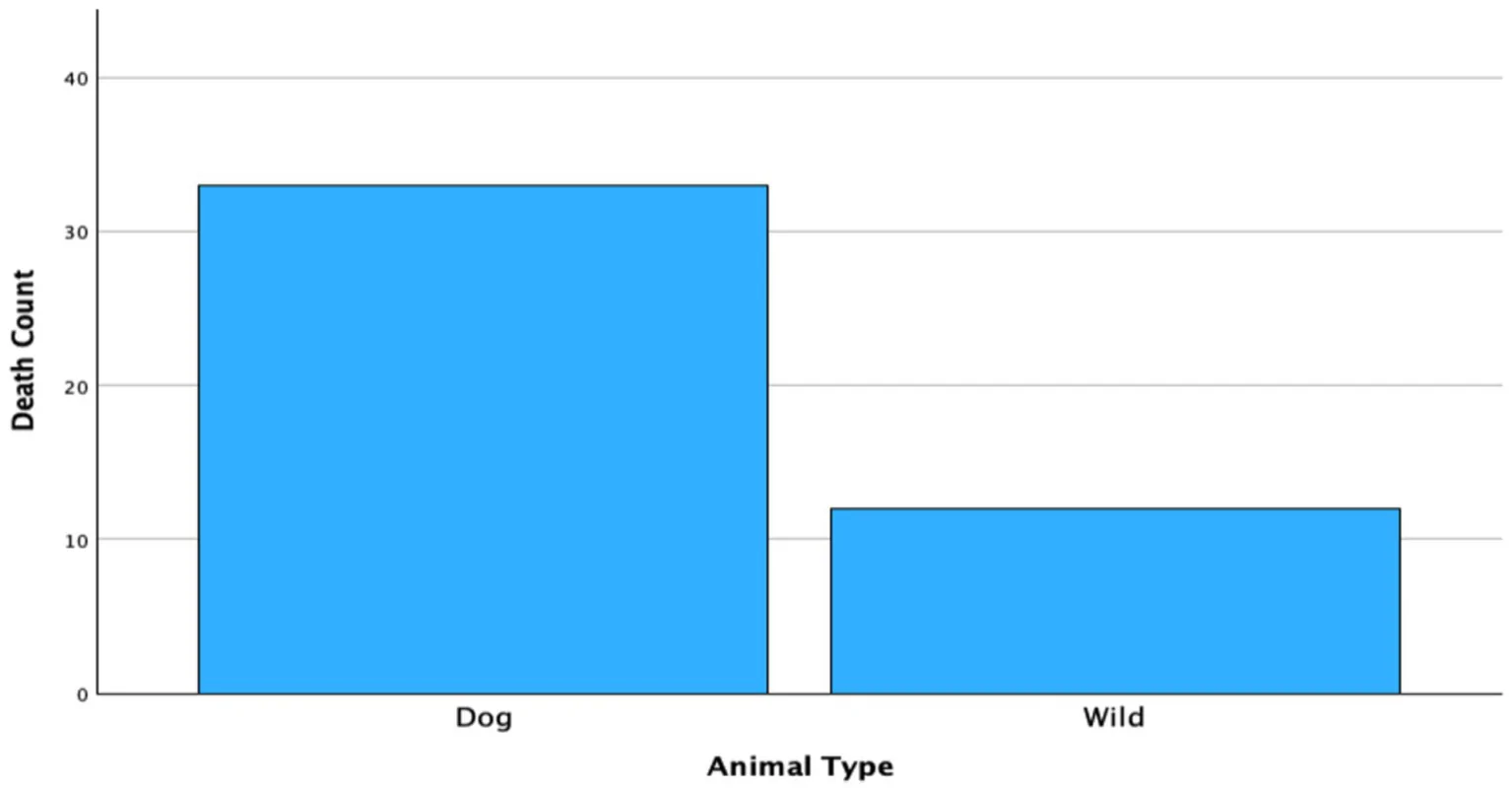
Fatalities due to animal exposures by animal type.

Importantly, no fatalities were reported among children or adolescents throughout the study period (Figure 9).

**Figure 10:**
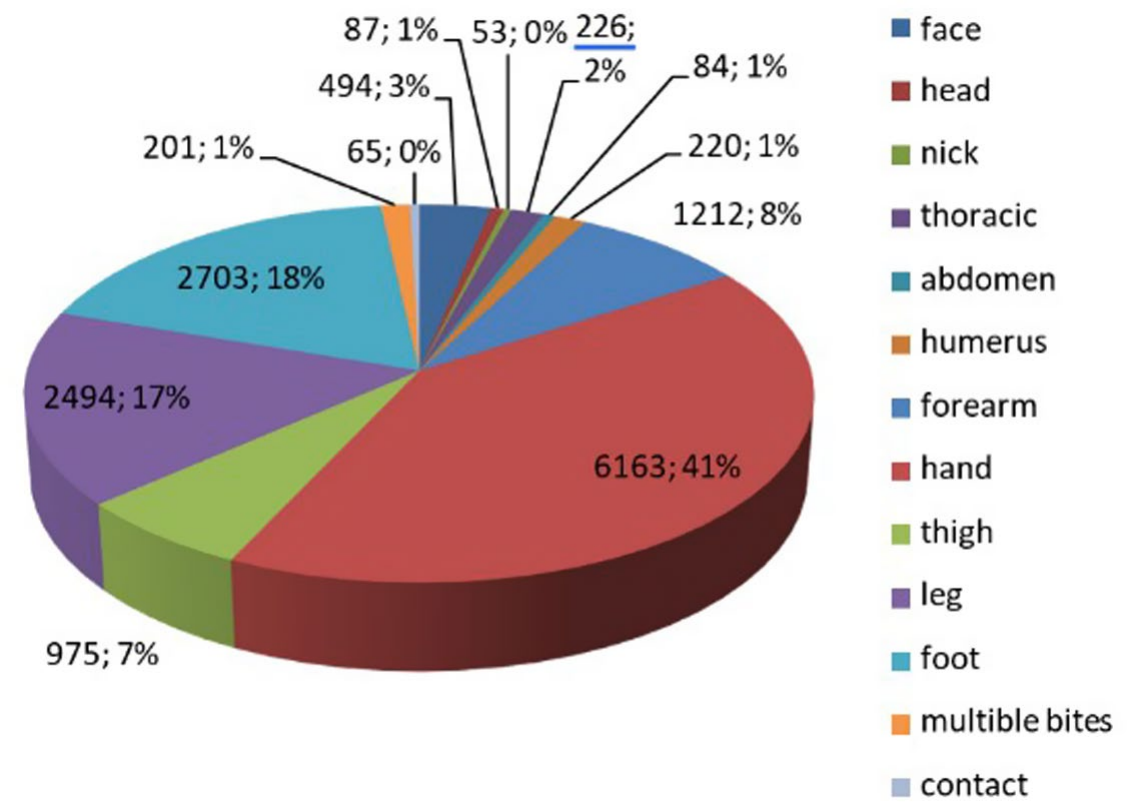
Location of the animal bite.

The authors observed a statistically significant difference in the anatomical distribution of animal bite injuries across body sites (χ^2^ = 27,654.968, df = 12, *p* < 0.01). The most frequently affected sites were the hands (41%), followed by the feet (18%) (Figure 10).

**Figure 11:**
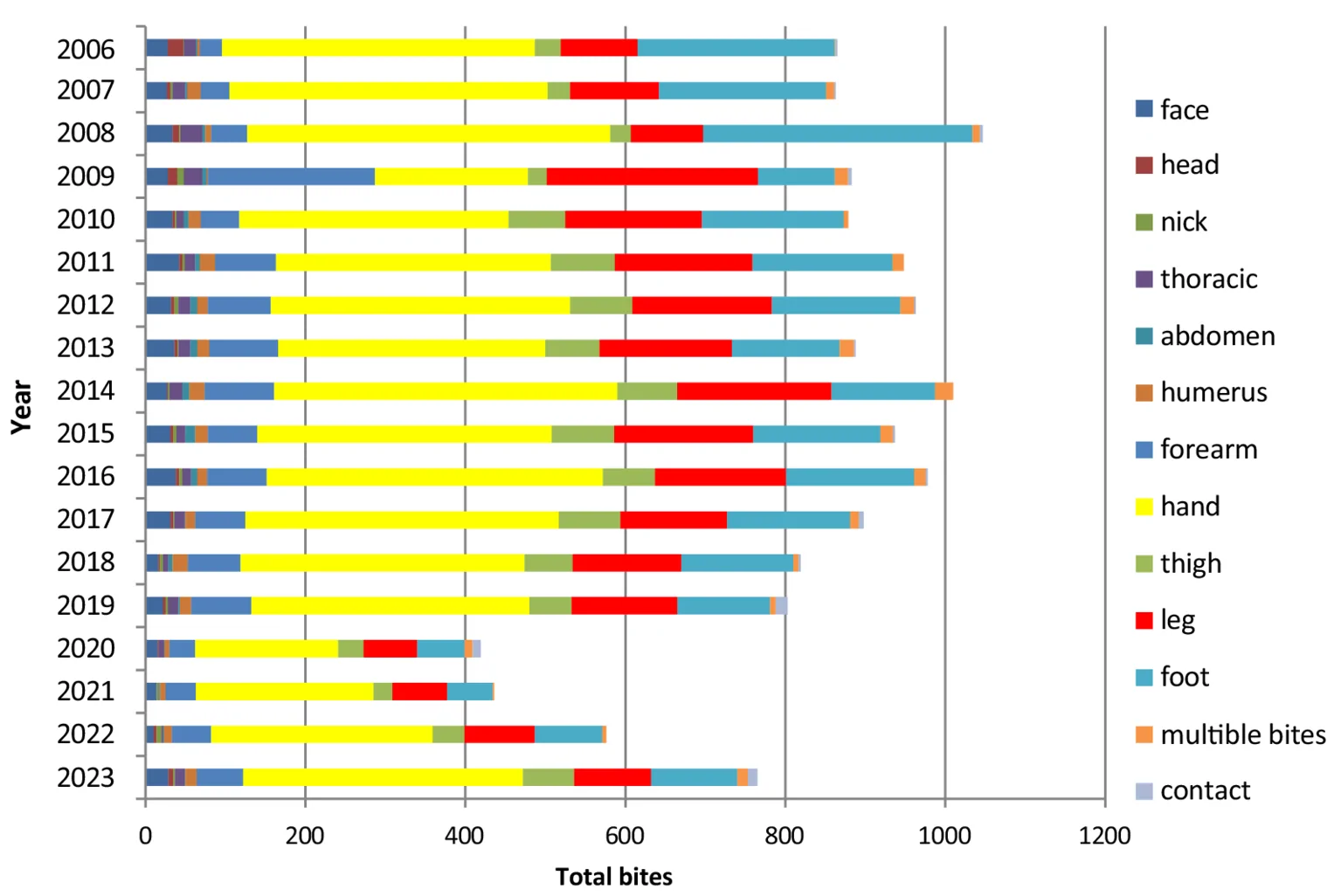
Location of animal bites by year.

In contrast, no statistically significant temporal variation in the anatomical location of animal bite injuries was observed across years, indicating that the distribution of bite locations remained stable over time (Figure 11).

The majority of animal bite incidents (88%) were caused by domestic animals, predominantly dogs, which accounted for 78.9% of all reported cases (Figure 9). Wild animals were responsible for 12% of incidents.

**Figure 12:**
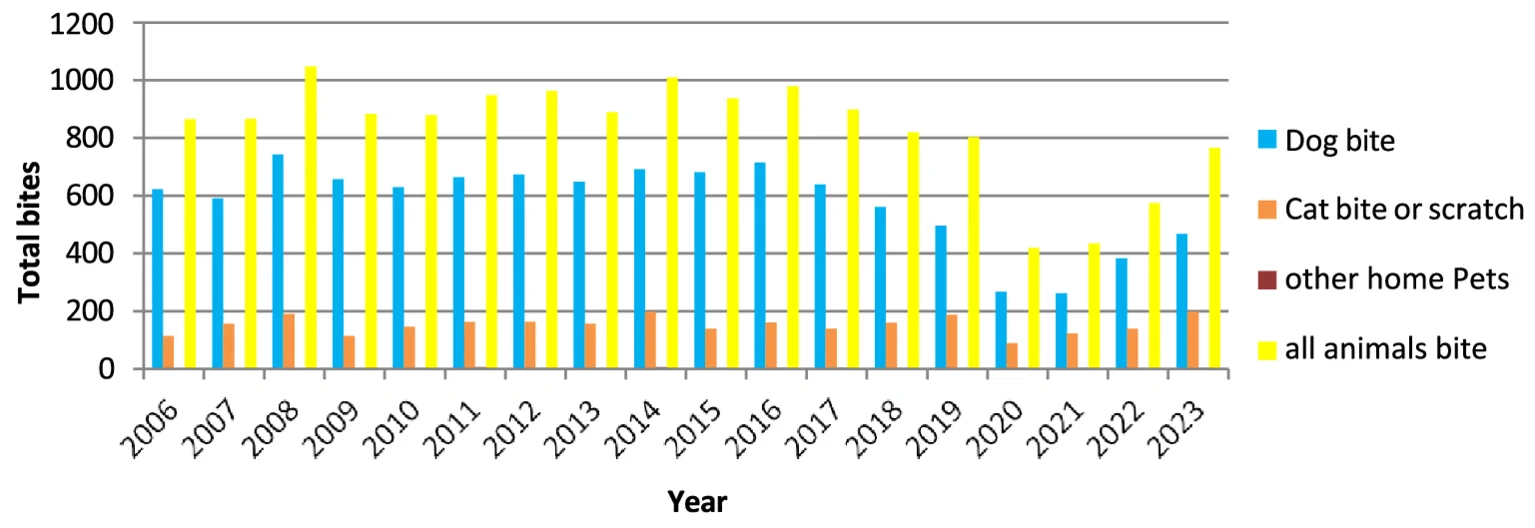
Relationship between dog, cat, and other home pets’ exposures and wild animal exposures.

Other domestic animals contributed only marginally to the overall number of cases; notably, horses accounted for just two cases (0.02%) over the 18-year observation period (Figure 12).

## 4 Discussion

To the best of our knowledge, this is the first study to comprehensively investigate animal bites and suspected rabies exposures in humans across all age groups at the national level in Slovakia. The dataset therefore provides a unique and nationally representative overview, owing to its extensive temporal coverage and large sample size.

Over a 37-year period (1987–2023), a total of 51,208 individuals were recorded as having been exposed to animals. Of these, 14,978 cases from 2006 to 2023 were included in detailed statistical analyses due to the availability of complete and standardised data. Importantly, no cases of human rabies have been reported in Slovakia during the past 33 years, with the last fatal case documented in 1990. This finding likely reflects the high effectiveness of Slovakia’s national rabies prevention programme, including systematic vaccination of domestic animals and the timely administration of post-exposure prophylaxis following suspected exposure prophylaxis, supporting the success of established public health measures (10).

The majority of animal bite incidents were attributed to dogs, accounting for 88% of pet-related exposures and 78.9% of all recorded animal exposures. These findings are consistent with reports from Turkey, the United States, and several European countries, which similarly identify dogs as the primary source of animal-related injuries (7, 11, 17, 18).

Our analysis further demonstrated a statistically significant association between animal species and calendar year, suggesting temporal changes in the pattern of animal exposures (Table 1). Although dogs consistently remained the predominant source of exposure, fatal outcomes were more frequently associated with wild animals. This observation may reflect a higher risk of rabies transmission, delayed access to medical care, or more severe injury mechanisms following attacks by wild animals. Notably, negligible number of animal rabies have been reported in Slovakia since 2006, further underscoring the effectiveness of national veterinary vaccination and rabies control programmes (11, 18).

In terms of demographics, the highest number of recorded cases of animal bite cases was observed among individuals aged 25–34 years, followed by those in the 35–44 and 45–54 age groups. Paediatric cases were also substantial, particularly among children aged 5–14 years, highlighting their vulnerability to animal-related injuries. These age-related patterns are consistent with findings from studies conducted in Texas (United States) and Australia, where children aged 2–9 years are frequently identified as high-risk populations for dog bite injuries (7, 16, 19).

Similar age-specific trends have been reported in Rome (2), Slovenia (13), and Australia (16), further reinforcing the need for early, age-appropriate educational interventions aimed at reducing animal bite risk.

Overall, this study underscores the importance of targeting both young children and working-age adults in prevention strategies aimed at reducing the incidence of animal bite injuries.

From an anatomical perspective, the distribution of injury locations provides important insight into injury severity and exposure patterns. The hand was the most frequently affected anatomical site, followed by the foot, and leg. These findings are consistent with studies conducted in Turkey, the United States, and Chile, which similarly report a predominance of extremity injuries resulting from animal bites. In contrast, some regions have documented a higher incidence of head and neck injuries, particularly among younger children (13, 14, 16, 17, 19, 20).

Our results align more closely with studies demonstrating a predominance of upper and lower limb injuries among adults, whereas craniofacial injuries are more commonly observed in paediatric populations. This distinction highlights the influence of age-related behavioural patterns, body height, and defensive responses during animal encounters.

All recorded fatalities occurred exclusively among adults, with the highest number observed in individuals aged ≥65 years. This finding is consistent with existing evidence indicating that older adults are at increased risk of severe complications and fatal outcomes following animal bite injuries (10).

Although fatalities were more frequent among males than females (34 vs. 11), chi-square analysis did not demonstrate a statistically significant association between sex and mortality (*p* = 0.079).

This observed trend may reflect differences in exposure patterns, occupational risks, or health-seeking behaviours between men and women. Similar sex-related patterns have been reported in previous studies, further supporting the potential role of socio-behavioural factors in determining bite-related outcomes.

Rabies vaccination data indicated a high level of post-exposure prophylaxis (PEP) coverage: 11,109 patients completed the full vaccination regimen, 1,395 received partial vaccination, and 2,258 did not receive vaccination. Importantly, no cases of human rabies were reported during the study period, underscoring the effectiveness of Slovakia’s national rabies control and PEP programmes (18, 19).

These findings are particularly noteworthy when contrasted with data from the United Kingdom, where approximately 62.3% of individuals sustaining dog bite injuries reportedly do not seek medical attention, potentially resulting in a substantial underestimation of the true national incidence of animal bite injuries (15). This comparison highlights the critical role of accessible healthcare services and public awareness in ensuring timely post-exposure management and accurate epidemiological surveillance.

Trend analysis and regression modelling demonstrated a significant decline in the incidence of animal bite injuries per 100,000 population over time (*β* = −0.507, *p* = 0.002). The lowest incidence rates were observed during the COVID-19 pandemic years (2020–2021), a finding that contrasts with reports from other settings, such as the study by Menk et al. (21), which documented an increase in dog bite incidents during lockdown periods.

These discrepancies may be explained by differences in national lockdown measures, reporting practices, and reduced outdoor human–animal interactions in Slovakia during the pandemic. The moderate increase observed in 2022–2023 likely reflects a post-pandemic return toward pre-pandemic levels of human–animal contact rather than a sustained upward trend. Slovakia’s successful elimination of human rabies is consistent with the strategic goals of the World Health Organization (7).

Nevertheless, animal bites—particularly dog bites—remain a substantial and largely preventable public health burden. The key findings of this study can be summarised as follows:

- High-risk groups: Children aged 5–9 years and adults aged 25–34 years exhibited the highest incidence of animal bite injuries, consistent with global epidemiological trends (8–11, 13–16). The predominance of males among fatal cases (*p* < 0.01) suggests a potential contribution of occupational exposure and sex-specific behavioural risk factors within the Slovak population.
- Post-pandemic trends: In contrast to reports of increased bite incidence during COVID-19 lockdowns in other countries (21), Slovakia experienced a distinct post-pandemic rebound, with an approximately 50% increase in cases during 2022–2023. This pattern highlights country-specific dynamics in the resumption of human–animal interactions following pandemic-related restrictions.
- Vaccination success: A high adherence rate to post-exposure prophylaxis (72.3%), together with the absence of human rabies cases since 1990, underscores the effectiveness of Slovakia’s national rabies prevention and vaccination policies.

### 4.1 Policy implications

Implement and enforce legislation, combined with mandatory owner training and certification, to reduce the risk of aggressive dog behaviour. Develop and support targeted educational campaigns aimed at high-risk groups, particularly school-based prevention programmes for children, to promote safe and responsible human–animal interactions.

### 4.2 Limitation of the study

This study has several limitations that should be acknowledged. First, underreporting of minor exposures—such as superficial bites, scratches, or drooling that did not require medical attention—is likely. The dataset included only individuals who sought medical care; therefore, the true incidence of animal-related injuries and suspected rabies exposures in Slovakia is likely underestimated.

Second, the absence of comprehensive data on stray dogs represents a potential limitation. However, this issue is relatively uncommon or largely absent in Slovakia due to established animal control and registration measures.

Additionally, only partial records were available for the period 1987–2005, and no data were available prior to 1987, which limited the assessment of long-term temporal trends. Another limitation is the lack of detailed behavioural and contextual information within the surveillance records, such as owner negligence, animal provocation, or the circumstances surrounding exposure events. Furthermore, the reliance on aggregated surveillance data restricted the application of multivariate analytical approaches and limited the assessment of individual-level clinical outcomes, including wound severity, treatment duration, and clinical progression.

Despite these limitations, the dataset provides a comprehensive and nationally representative overview of medically attended animal exposures and suspected rabies cases in Slovakia over an extended observation period.

## 5 Conclusion

This study provides the first comprehensive national analysis of animal bite incidents and suspected rabies exposures in Slovakia over an 18-year period (2006–2023). Dog bites accounted for approximately 80% of all reported incidents, with children and adolescents comprising the most affected age group, dog bites represent a major public health issue in Slovakia. A marked increase in bite incidence was observed following the COVID-19 lockdown period, with rates rising by nearly 50%. The most frequent anatomical locations of injury were the hands (41%), followed by the feet (18%). No human rabies cases were recorded during the study period, confirming the continued effectiveness of Slovakia’s rabies elimination programme. However, fatal attacks occurred predominantly among older men and were associated primarily with wild animals, underscoring ongoing risks in rural and forested regions.

These findings highlight the need for targeted, evidence-based prevention strategies. Public education on safe human–animal interactions should be prioritised for children and adolescents, who bear the greatest burden of injuries. Enhanced adult supervision, responsible pet ownership, and zero-tolerance policies toward aggressive domestic animals are critical components of prevention. The post-pandemic increase in bite incidents also suggests that public health systems should anticipate and mitigate indirect consequences of societal disruptions. Further research is warranted to better understand the influence of animal behaviour and ownership practices on bite incidents, and to optimise prevention strategies for high-risk groups, including young children and older adults in rural areas.

From a broader perspective, Slovakia’s rabies control programme represents a successful model of One Health policy implementation.

## Data Availability

The original contributions presented in the study are included in the article/supplementary material, further inquiries can be directed to the corresponding author.
